# Supplementary material for: Universal scaling of the self-field critical current in superconductors: from sub-nanometre to millimetre size
Source: Sci Rep. 2017 Aug 30;7:10010. doi: 10.1038/s41598-017-10226-z (PMC5577115; doi:10.1038/s41598-017-10226-z)
Supplement: Supplementary file 1 — Supplementary Information [file 41598_2017_10226_MOESM1_ESM.pdf]

Supplementary Information: Universal scaling of the self-field critical current in superconductors: from sub-nanometre to millimetre size

E. F. Talantsev[1], W. P. Crump[1] and J. L. Tallon[1,2]
[1] Robinson Research Institute, Victoria University of Wellington, P.O. Box 33436, Lower Hutt 5046, New Zealand.
[2] MacDiarmid Institute for Advanced Materials and Nanotechnology, P.O. Box 33436, Lower Hutt 5046, New Zealand.

1. Edge field for a rectangular superconductor at J_c

We adopt the same geometry shown in Fig. 1 of our MS. The *y*-component, B_y, of the edge field at $(x,y) = (\pm a,0)$ is calculated by Brojeny and Clem [1] under the assumption of uniform current density to be

$$B_y = (\mu_0 J_c b/\pi)\left[\ln\left(\frac{2a}{b}\right) + 1\right] \quad \text{(S1)}$$

Replacing J_c by J_s using Eq.(6) in the MS, namely

$$J_c = J_s(\lambda/b)\tanh(b/\lambda)$$

and making the further replacement

$$J_s = B_{c1}/(\mu_0\lambda)$$

we have

$$B_y = (B_{c1}/\pi)\tanh(b/\lambda)\left[\ln\left(\frac{2a}{b}\right) + 1\right] \quad \text{(S2)}$$

While this is expressed for all b it is, strictly, only for uniform current density i.e. when $b < \lambda$. However, as noted in the MS we believe this formula is valid also for $b > \lambda$. To see this we consider replacing the rectangular conductor by an array of round wires each of radius b, of width $2a$, as shown in Fig. S1. There are thus N such wires, where $N = a/b$.

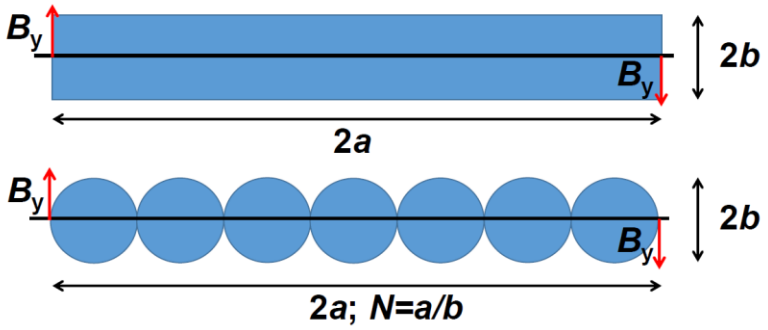

Fig.S1. The rectangular conductor of cross-section $2a \times 2b$ is approximated by an array of N round wires, each of radius b and total width $2a$ so that $N = a/b$.

Because of the smaller profile area we scale up the total current in proportion to the area. Thus J_c(array) = $(4/\pi)$ J_c(rectangle). Using Ampere's law we can calculate exactly the net

field B_y at the edges arising from each current carrying wire. And we can do so for any value of b whether the current distribution is uniform ($b << \lambda$) or just confined to the surface ($b >> \lambda$) – we need only know the total current flowing within each round wire. Summing up each contribution we have:

$$B_y = (\mu_0 J_c b/2) \sum_{n=1}^{N=a/b} \frac{1}{2n-1} \tag{S3}$$

The sum can be evaluated as follows. The sum of $1/n$ from n=1 to $n = 2N$ can be broken into the sum of odd terms (which is what we want) and the sum of even terms:

$$\sum_{n=1}^{2N} \frac{1}{n} = \sum_{n=1}^{N} \frac{1}{2n-1} + \frac{1}{2}\sum_{n=1}^{N} \frac{1}{n}$$

Then noting that:

$$\sum_{n=1}^{N} \frac{1}{n} \xrightarrow[n\to\infty]{} \ln(N) + \gamma$$

Where γ is Euler's constant, γ=0.57722. Thus replacing N by a/b Eq.(S3) becomes:

$$B_y = (\mu_0 J_c b/\pi)\left[\ln\left(\frac{2a}{b}\right) + \ln(2) + \gamma\right]$$

Or replacing J_c by J_s (λ/b).tanh(b/λ) = $B_{c1}/(\mu_0\lambda)$.(λ/b).tanh(b/λ) we have finally:

$$\boxed{B_y = (B_{c1}/\pi)\tanh(b/\lambda)\left[\ln\left(\frac{2a}{b}\right) + 1.2704\right]} \tag{S4}$$

This is almost identical to Eq.(S2) but it applicable for all b. We therefore conclude that Eq.(S2) is itself applicable for all b.

2. Scaling of J_c(sf) for anisotropic superconductors

Consider an anisotropic superconducting film with axes as described in Fig. 1 of the MS where the crystallographic c-axis is normal to the film i.e. aligned along the y-direction. Solving Londons' equations for the current distribution along the y-axis we obtain:

$$J_c = J_s \times (\lambda_c/b)\tanh(b/\lambda_c) \quad , \tag{S5}$$

where λ_c is the c-axis penetration depth. This replaces Eq. (2) in the MS. Accordingly the scaling equation for the normalised J_c is

$$J_c^n = (\lambda_c/b)\tanh(b/\lambda_c) \quad . \tag{S6}$$

The normalising factor is $J_s = B_{c1}/(\mu_0\lambda)$ as before i.e. using only $\lambda = \lambda_{ab}$ with no contribution from λ_c. Clearly the effects of anisotropy disappear when b << λ_c. The equation for the normalised B_s is

$$B_s^n \equiv B_x^n = \gamma.\tanh(b/\lambda_c)$$

, (S7)

where $\gamma = \lambda_c/\lambda_{ab}$ is the anisotropy parameter, and the equation for the normalised edge field is

$$B_y^n = (\gamma/\pi).\tanh(b/\lambda_c)\,[\ln(2a/b) + 1] \quad . \qquad (S8)$$

Note that, as for J_c^n, the effects of anisotropy disappear for both the surface field and the edge field when $b \ll \lambda_c$. And over the entire range of b we see that J_c^n follows the same scaling behaviour as that for isotropic systems provided the thickness is scaled as (b/λ_c). The normalised surface field only scales identically with isotropic systems if one plots B_s^n/γ versus (b/λ_c) and as before the normalised edge field does not scale at all because of the $\ln(2a/b)$ term. Eq. (S6) thus provides a means of directly determining the anisotropy factor γ by plotting J_c^n versus (b/λ_{ab}). The knee in the black curve shown in Figs. 3 and 4 of the MS will be displaced to the right of (b/λ_{ab}) = 1 by the factor γ. This may be found by extrapolating the linearly-falling $1/b$ behavior in the log-log plot back to $J_c^n = 1$. The abscissa is then γ. We illustrate this in the MS for the case of YBCO where we find in this way $\gamma = 7$.

3. Raw plot of J_c versus b prior to scaling

In Fig. S2 we show the raw J_c data plotted vs b (or a for round wires) prior to the scaling shown in Fig. 3 of our MS. This shows the broad scatter in the data but already, for large b, shows the fall off reflecting the fact that the current flow is confined only to the London screening layer and not the full cross-section. Orange data points are for ionic-liquid gated YBCO at different doping as discussed in section 3.

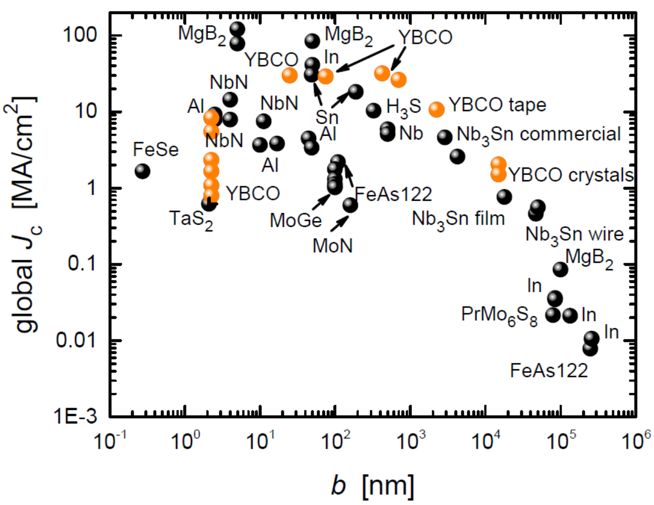

Fig. S2. Plot of raw J_c(sf) data versus film half-thickness b (or a for round wires).

4. Plot of raw J_c versus b/λ

Fig. S3 now shows the raw J_c data plotted vs b/λ (or a/λ for round wires). This still shows the broad scatter in the data prior to full scaling.

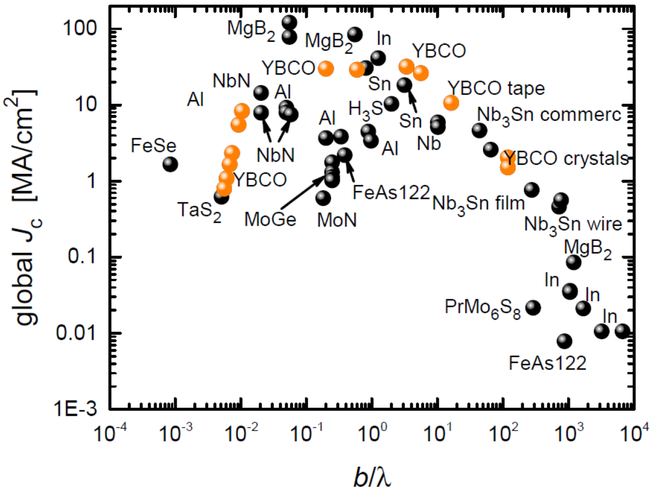

Fig. S3. Plot of raw J_c(sf) data versus scaled film thickness b/λ (or a/λ for round wires).

5. Scaling plot including doping dependence of J_c for $YBa_2Cu_3O_{7-\delta}$

$YBa_2Cu_3O_{7-\delta}$ (YBCO) has a variable hole doping state depending on δ or cation doping. As the doping progresses from underdoped to slightly overdoped YBCO progresses from weak superconductivity to strong superconductivity with a marked increase in critical fields and in J_c. Fête *et al.* [2] have used ionic liquid gating to change the doping state of 4-5 nm thick YBCO films and measure T_c and J_c , using our analysis to calculate the penetration depth and superfluid density. They find a Uemura plot of T_c versus superfluid density which well matches other independent measures using muon spin relaxation. We plot their data in Fig. S2, S3 and S4 by the string of orange data points near b = 2.25 nm or $(b/\lambda) \approx 10^{-2}$, using the Uemura plot reported in [3] to determine the magnitude of λ for each doping state. The scaled data is plotted below in Fig. S4 and the string of data points for YBCO fit the scaling plot rather well. Other YBCO data points in the plot are also presented in orange for ease of comparison. As b increases and then exceeds λ (in this case λ_c) we see the generic crossover from $J_c \propto \lambda^{-3}$ to $J_c \propto \lambda^{-2} b^{-1}$ in a single superconductor.

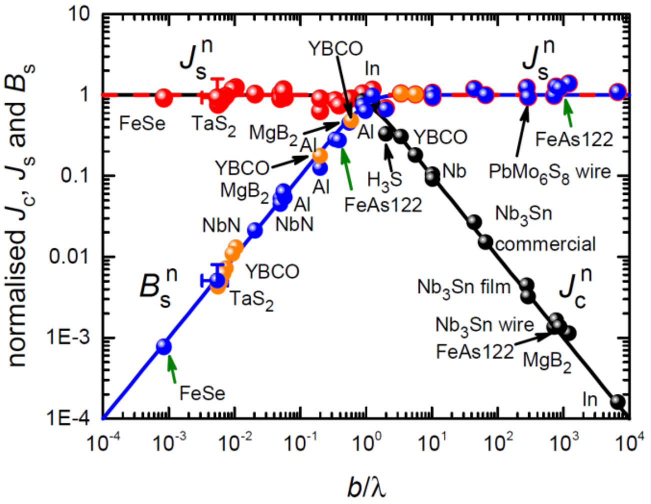

Fig. S4. Scaling plot of normalised J_c, B_s and J_s showing data for YBCO highlighted in orange for the B_s^n data. The string of data points around $(b/\lambda) \approx 10^{-2}$ are obtained from ionic liquid gating [2], where the doping is changed by field effect.

6. Scaling plot including temperature dependence

The scaling plot in the MS uses ground state values only of J_c, B_s and J_s. All of the data sets used are T-dependent data sets from which the ground state values were determined by fitting and extrapolation. However the same analysis can be applied at any temperature and we show in Fig. S5 two additional T-dependent data sets (we omit the red J_s^n data points for clarity). Firstly, at the extreme left is shown the $J_c(T)$ data for single atomic layer FeSe [4]. While b remains fixed the value of λ rises with increasing T. Accordingly the value of (b/λ) falls as shown by the five data points extending down to $(b/\lambda) = 10^{-4}$. This now extends our scaling to eight orders of magnitude in (b/λ).

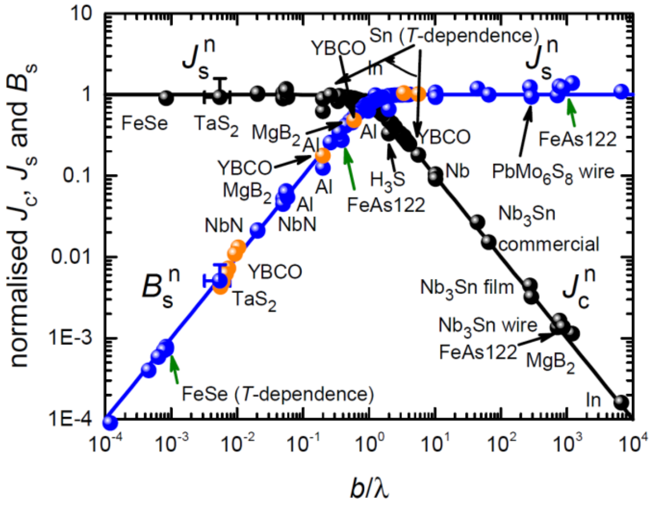

Fig. S5. Scaling plot of normalised J_c, B_s and J_s showing the effect of T-dependence in J_c for single-atom-layer FeSe [4] (annotated) and for Sn [5] (annotated). In each case (b/λ) decreases as λ increases with T. The Sn data [5] situated around $b \approx \lambda$ reveals the generic crossover from $J_c \propto \lambda^{-3}$ to $J_c \propto \lambda^{-2} b^{-1}$.

Secondly, around the middle of the plot is the $J_c(T)$ data of Song [5] for Sn (the data is taken from the thesis of Song). This shows in a single sample and a single experiment the crossover from $J_c \propto \lambda^{-3}$ to $J_c \propto \lambda^{-2} b^{-1}$ arising because λ increases with increasing T. Thus with increasing T the data points progress to the left. Those authors observed a change in T-dependence which they interpreted as a crossover to Josephson-junction-like behavior, but the change is precisely as expected in the function $(\lambda/b)\tanh(b/\lambda)$ when b falls less than λ.

References:

[1] Brojeny, A.B. and Clem, J.R. Self-field effects upon the critical current density of flat superconducting strips. Supercond. Sci. Technol. **18**, 888-895 (2005).
[2] Fête, A., Rossi, L., Augieri, A. and Senatore, C. Ionic liquid gating of ultra-thin $YBa_2Cu_3O_{7-x}$ films, Appl. Phys. Lett. **109**, 192601 (2016).
[3] Tallon, J.L., Loram, J.W. & Cooper, J.R. The superfluid density in cuprate high-T_c superconductors – a new paradigm. Phys. Rev. B **68**, 180501 (R) (2003).
[4] Zhang, W.-H. *et. al*., Direct observation of high-temperature superconductivity in one-unit-cell FeSe films. Chinese Phys. Lett. **31**, 017401 (2014).
[5] Song, Y.-D. and Rochlin, G.I. Transition from bulk-like behavior to Josephson-junction-like behavior in superconducting microbridges, Phys. Rev. Lett. **29**, 416 (1972).
[6] Talantsev, E.F. and Tallon, J.L. Universal self-field critical current for thin-film superconductors. Nature Comms. **6**, 7820-7827 (2015).
